# Perioperative stroke and survival in coronary artery bypass grafting patients: a SWEDEHEART study

**DOI:** 10.1093/ejcts/ezac025

**Published:** 2022-01-25

**Authors:** Kristjan Jonsson, Mikael Barbu, Susanne J Nielsen, Brynhildur Hafsteinsdottir, Tomas Gudbjartsson, Elin M Jensen, Martin Silverborn, Anders Jeppsson

**Affiliations:** 1 Department of Molecular and Clinical Medicine, Institute of Medicine, Sahlgrenska Academy, University of Gothenburg, Gothenburg, Sweden; 2 Department of Cardiothoracic Surgery, Sahlgrenska University Hospital, Gothenburg, Sweden; 3 Department of Cardiology, Blekinge Hospital, Karlskrona, Sweden; 4 Department of Neurology, Sahlgrenska University Hospital, Gothenburg, Sweden; 5 Faculty of Medicine, University of Iceland, Reykjavik, Iceland; 6 Department of Cardiothoracic Surgery, Landspitali, Reykjavik, Iceland

**Keywords:** Coronary artery bypass grafting, Perioperative stroke, Incidence, Mortality

## Abstract

**OBJECTIVES:**

Perioperative stroke is a severe complication of cardiac surgery. We assessed the incidence of stroke over time, the association between stroke and mortality and identified preoperative factors independently associated with perioperative stroke, in a large nationwide cardiac surgery population.

**METHODS:**

All patients who underwent coronary artery bypass grafting in Sweden 2006–2017 were included in a registry-based observational cohort study based on prospectively collected data. Multivariable logistic and Cox regression models were used to assess associations between perioperative stroke and mortality and to identify factors associated with stroke. The median follow-up was 6 years (range 0–12).

**RESULTS:**

There were 441 perioperative strokes in 36 898 patients. The mean incidence was 1.2% and decreased marginally over time [adjusted odds ratio (OR) 0.97 per year (95% confidence interval 0.94–1.00), *P* = 0.035]. Stroke patients had a higher overall mortality risk during follow-up [adjusted hazard ratio 2.30 (2.00–2.64), *P* < 0.001], with the highest risk during the first 30 postoperative days [adjusted hazard ratio (7.29 (5.58–9.54), *P* < 0.001]. The strongest independent preoperative factors associated with stroke were prior cardiac surgery [adjusted OR 2.89 (1.40–5.96)], critical preoperative condition [adjusted OR 2.55 (1.73–3.76)], previous stroke [adjusted OR 1.77 (1.35–2.33)], preoperative angina requiring intravenous nitrates [adjusted OR 1.67 (1.28–2.17)], peripheral vascular disease [OR 1.63 (1.25–2.13)] and advanced age [OR 1.05 (1.03–1.06) per year].

**CONCLUSIONS:**

The incidence of perioperative stroke after coronary artery bypass grafting has remained stable. Patients with perioperative stroke had a markedly higher adjusted risk of death early after surgery. The risk declined over time but remained higher during the entire follow-up period.

**Figure:**
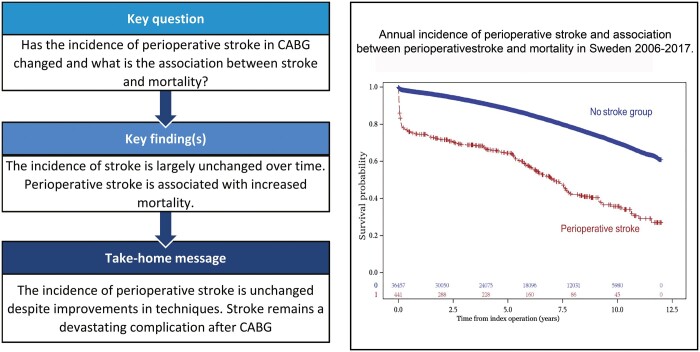

## INTRODUCTION

Perioperative stroke is one of the most devastating complications in cardiac surgery. The incidence of perioperative stroke varies in different studies depending on the study population and stroke definition. In large registry-based studies, the incidence of perioperative stroke varies in mixed cardiac surgery populations from 1.6% to 4.6% and in coronary artery bypass grafting (CABG) patients from 1.1% to 5.7% [1–7]. A recent meta-analysis of mixed cardiac surgery patients reported an incidence of 2.0% [5].

While several studies have reported on the strong association between perioperative stroke and early mortality after cardiac surgery [8, 9], there is less information about the association between perioperative stroke and long-term mortality risk in cardiac surgery patients. Furthermore, most previous investigations on the subject are either limited in size and/or by single-centre design. Likewise, studies on preoperative factors associated with perioperative stroke are attributed to the same limitations. Hence, in the present population-based study, based on a large whole nationwide cohort with complete long-term follow-up, our objectives were to assess the incidence of perioperative stroke over time, identify preoperative factors independently associated with perioperative stroke and assess the association between stroke and short- and long-term mortality.

## METHODS

### Ethics statement

The study was approved by the Regional Ethics Committee in Gothenburg, Sweden (registration number 139-16 approved 4 April 2016). The Ethics Committee waived individual patient consent due to the registry-based study design. The study was performed in accordance with the 1975 Declaration of Helsinki. Before the analysis, all personal identifiers were replaced by codes to ensure anonymity.

### Data availability statement

The data underlying this article were provided by the Swedish Web System for Enhancement and Development of Evidence-Based care in Heart disease Evaluated According to Recommended Therapies (SWEDEHEART) and national healthcare registries in Sweden. Data will be shared on request to the corresponding author with permission of SWEDEHEART and The National Board of Health and Welfare.

### Study design and study population

In this nationwide, population-based cohort study, the study population was identified in the Swedish Heart Surgery Registry, which is a part of the SWEDEHEART registry [11]. All patients >18 years who underwent isolated CABG in Sweden from 1 January 2006 until 31 December 2017 were included. Patients who died on the day of surgery were excluded due to uncertain diagnosis of intraoperative stroke. Also, patients who had missing data for perioperative stroke in the registry were excluded. Patients who emigrated during the follow-up period were censored at the time of emigration. The manuscript has been composed according to recommendations in the Strengthening the Reporting of Observational Studies in Epidemiology statement [10].

### Data sources and definitions

Data from 3 national registers were merged through the personal social security number, unique for all Swedish citizens. The SWEDEHEART registry has a national coverage of 99% [11] and contains information about pre-, intra- and postoperative clinical information for all patients who have undergone cardiac surgery in Sweden since 1992. Perioperative stroke is defined in the SWEDEHEART registry as any focal neurological deficit occurring postoperatively, during the index hospitalization, and lasting >72 h. The diagnosis in the registry is based on clinical symptoms and does not necessarily require confirmation with imaging studies. The European System for Cardiac Operative Risk Evaluation I definitions were used for critical preoperative status and unstable angina [12].

Diagnosis for the patients’ medical history, other than in the SWEDEHEART registry, was retrieved from The National Patient Register. Registration of principal and contributory diagnoses for all hospitalizations in Sweden is mandatory. The registry has complete national coverage for all hospitalized patients since 1987 with a validity of 85–95% [13]. The International Statistical Classification of Diseases system (ICD) was used to identify the diagnoses: ICD 9 for the period 1987–1997 and ICD 10 for the period 1997–2017 (Supplementary Material, Table S1). Information about mortality was collected from the national Cause of Death register [14], which has information on the date and cause of death of all deceased Swedish citizens.

### Statistical analyses

Descriptive continuous variables are presented by mean and standard deviation and categorical variables by frequencies and percentages. For comparison between 2 groups, Fisher’s exact test (lowest one-sided *P*-value multiplied by 2) was used for dichotomous variables.

Univariable and multivariable logistic regression models were used to study the annual trends of the incidence of perioperative stroke and to identify factors associated with perioperative stroke. The full adjustment of the annual trends included age, sex, previous ischaemic stroke, previous transient ischaemic attack, previous haemorrhagic stroke, previous myocardial infarction, diabetes, heart failure, hypertension, atrial fibrillation, renal failure, peripheral vascular disease, history of cancer, chronic respiratory disease, acute coronary syndrome, previous heart surgery and angina at rest. Factors with a *P*-value of <0.05 in the univariable model were considered for inclusion in the multivariable model using backward selection. Age and sex were forced into the multivariable logistic model. Odds ratio (OR) with 95% confidence interval (CI) are presented from the logistic regression models. Interaction between sex and following variables was studied *posthoc*: age (<70 and ≥70 years), previous stroke, preoperative critical state and previous heart surgery.

The change in incidence of perioperative stroke over calendar time was assumed to be linear and tested using logistic regression. Hosmer–Lemeshow test was performed to test goodness-of-fit.

Cox regression models were used to compare mortality, between patients diagnosed with perioperative stroke and those with no perioperative stroke, within 30 days and beyond 30 days, which was divided into 3 time periods, 30 days to 1 year, 1 year to 5 years and >5 years. An overall estimate of HR for the whole time period was presented for completeness and should be interpreted as a mean HR over the studied time. The proportional hazards assumption was investigated by studying the interaction between the status of the perioperative stroke and log(follow-up time), which was found not to be satisfied for the periods 0–30 days and 30 days to 1 year. Further time splitting was therefore performed as follows: 0–7 days, 7 days to 30 days, 30 days to 3 months and 3 months to 1 year. Hazard ratio (HR) with 95% CI were presented from the Cox regression models. Survival probability was presented using the Kaplan–Meier technique.

All tests were two-sided and a *P*-value <0.05 was considered significant. All analyses were performed by using SAS Software version 9.4 (SAS Institute Inc., Cary, NC, USA).

## RESULTS

### Patients

A total of 37 173 patients underwent isolated CABG in Sweden during the study period. After exclusion of patients who died during the operation day (*n* = 48, 0.1%) and patients with missing information about perioperative stroke (*n* = 227, 0.6%), a total of 36 898 patients were included in the study. Out of these, 173 patients (0.47%) emigrated during follow-up and were censored at the time of emigration. Perioperative stroke occurred in 441 patients (1.2%). The stroke population was older, more likely to be female, and had a higher burden of comorbidities, including e.g. diabetes, previous stroke and peripheral artery disease, than the non-stroke group. The *posthoc* analyses revealed a significant interaction between sex and age category (*P* = 0.049) with larger difference in relative odds for female versus male in <70-year-old patients [OR 2.05 (95% CI 1.40–3.00)] than in ≥70-year-old patients [OR 1.30 (95% CI 1.01–1.66)]. No significant interaction was observed for previous stroke, preoperative critical state and previous heart surgery. Stroke patients had also higher logistic European System for Cardiac Operative Risk Evaluation I and CHA_2_DS_2_-VASC score. Patient characteristics in the stroke and no-stroke groups are presented in Table 1. The median follow-up was 6.0 years (range 0–12).

**Table 1:** Baseline characteristics in stroke and no-stroke patients

Preoperative variables	Missing data	Stroke, *n* = 441	No stroke, *n* = 36 457	*P*-Value
Mean age (years)	0 (0)	72 ± 8	68 ± 9.	<0.001
Male sex	0 (0)	315 (71.4)	29 306 (80.3)	<0.001
Body mass index (kg/m^2^)	2922 (7.9)	27.5 ± 4.2	27.5 ± 4.2	1.0
Logistic EuroSCORE I	120 (0.3)	6.6 ± 3.3	4.5 ± 2.9	<0.001
CHA_2_DS_2_-VASC score	0 (0)	3.9 ± 1.7	2.9 ± 1.7	<0.001
Diabetes	0 (0)	155 (35.1)	10 121 (27.7)	0.001
Previous myocardial infarction	0 (0)	247 (56.0)	17 759 (48.7)	0.002
Acute coronary syndrome	0 (0)	288 (65.3)	21 848 (59.9)	0.060
Renal failure	0 (0)	30 (6.8)	1222 (3.5)	0.001
Heart failure	0 (0)	114 (25.8)	5152 (14.1)	<0.001
Hypertension	0 (0)	299 (67.8)	21 624 (59.3)	0.014
Previous stroke	0 (0)	65 (14.7)	2499 (6.8)	<0.001
Atrial fibrillation	0 (0)	72 (16.3)	3360 (9.2)	<0.001
Chronic respiratory disease	629 (1.7)	40 (9.3)	2719 (7.6)	0.24
History of cancer	0 (0)	75 (17.0)	4814 (13.2)	0.028
Peripheral vascular disease	0 (0)	70 (15.9)	2930 (8.0)	<0.001
Hyperlipidaemia	0 (0)	150 (34.0)	12 722 (35.5)	0.40
Prior cardiac surgery	281 (0.7)	40 (9.1)	192 (0.5)	<0.001
Critical preoperative state	280 (0.7)	32 (7.3)	784 (2.2)	<0.001

Data are presented as numbers and percentages or mean ± SD.

EuroSCORE: European System for Cardiac Operative Risk Evaluation; SD: standard deviation.

### Incidence of stroke over time

**Figure 1:**
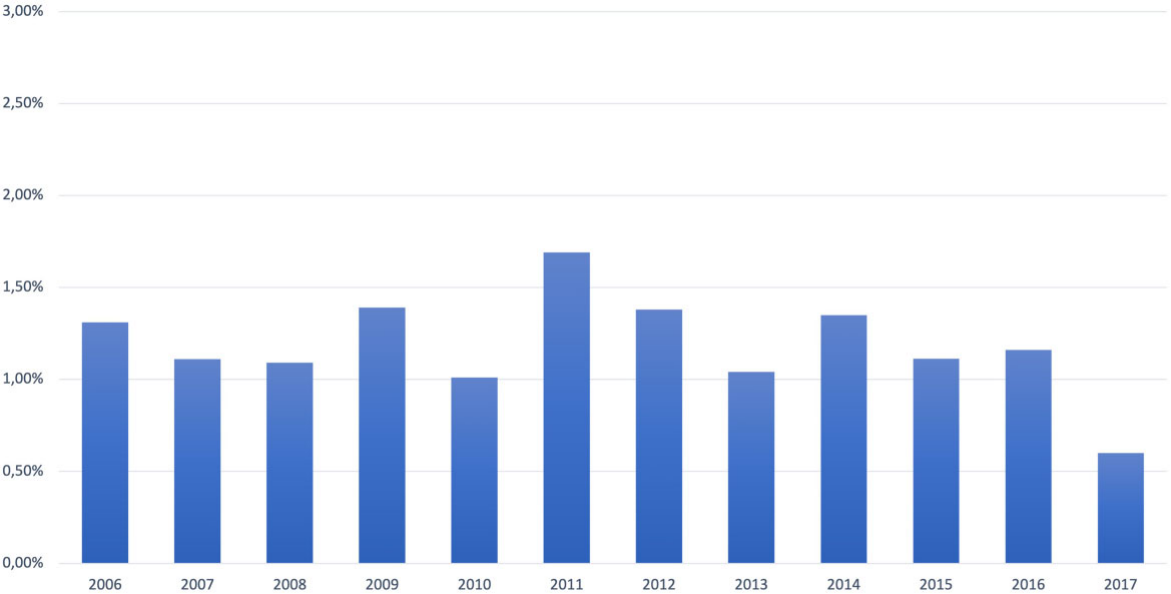
The annual incidence of perioperative stroke in coronary artery bypass grafting patients in Sweden 2006–2017.

The mean annual incidence of perioperative stroke was 1.2%. The annual incidence varied during the study period between 0.6% and 1.7% (Fig. 1). In the unadjusted and age-and-sex only adjusted logistic regression models, there was no significant change in the incidence of perioperative stroke over time [OR 0.98 (95% CI 0.96–1.01), *P* = 0.17 and 0.98 (95% CI 0.96–1.01), *P* = 0.19, respectively]. In the multi-adjusted model, there was a statistically significant but marginal reduction in the incidence of perioperative stroke over time [adjusted OR: 0.97 (95% CI 0.94–1.00), *P* = 0.035] (Hosmer–Lemeshow goodness-of-fit test *P* = 0.17).

### Preoperative factors independently associated with perioperative stroke

Preoperative factors independently associated with stroke are presented with ORs in Table 2. Prior to cardiac surgery, critical preoperative condition, previous stroke, angina at rest requiring intravenous nitrates, peripheral vascular disease, heart failure, female sex, atrial fibrillation, diabetes and age were independently associated with an increased risk for perioperative stroke.

**Table 2:** Factors associated with perioperative stroke in univariable and multivariable logistic regression models

Variable		Number of strokes (%)	Univariable analysis		Multivariable analysis	
			OR (95% CI)	*P*-Value	OR (95% CI)	*P*-Value
Sex, *n* (%)	Male	315 (1.1)	1.63 (1.33–2.01)	<0.001	1.39 (1.12–1.72)	0.003
	Female	126 (1.7)				
Age (years), mean ± SD	Stroke group	72.2 ± 7.6	1.06 (1.04–1.07)	<0.001	1.05 (1.03–1.06)	<0.001
	No-stroke group	68.2 ± 9.1				
Body mass index (kg/m^2^), mean ± SD	Stroke group	27.5 ± 4.2	1.00 (0.98–1.03)	0.81		
	No-stroke group	27.4 ± 4.2				
Hyperlipidaemia, *n* (%)	Yes	150 (1.2)	0.96 (0.79–1.17)	0.71		
	No	291 (1.2)				
Hypertension, *n* (%)	Yes	299 (1.4)	1.45 (1.18–1.77)	<0.001		
	No	142 (0.9)				
Diabetes, *n* (%)	Yes	155 (1.5)	1.41 (1.16–1.72)	<0.001	1.28 (1.04–1.56)	0.019
	No	286 (1.1)				
Previous stroke, *n* (%)	Yes	65 (2.5)	2.35 (1.80–3.07)	<0.001	1.77 (1.35–2.33)	<0.001
	No	376 (1.1)				
Previous transient ischaemic attack, *n* (%)	Yes	43 (2.1)	1.81 (1.32–2.49)	<0.001		
	No	398 (1.1)				
Previous myocardial infarction, *n* (%)	Yes	247 (1.4)	1.34 (1.11–1.62)	0.002		
	No	194 (1.0)				
Heart failure, *n* (%)	Yes	104 (2.2)	2.08 (1.67–2.60)	<0.001	1.54 (1.22–1.95)	<0.001
	No	337 (1.1)				
Prior cardiac surgery, *n* (%)	Yes	8 (4.0)	3.51 (1.72–7.16)	<0.001	2.89 (1.40–5.96)	0.004
	No	430 (1.2)				
Atrial fibrillation, *n* (%)	Yes	72 (2.1)	1.93 (1.49–2.49)	<0.001	1.34 (1.02–1.75)	0.032
	No	369 (1.1)				
Renal failure, *n* (%)	Yes	30 (2.4)	2.11 (1.45–3.07)	<0.001		
	No	411 (1.2)				
Peripheral vascular disease, *n* (%)	Yes	70 (2.3)	2.17 (1.67–2.80)	<0.001	1.63 (1.25–2.13)	<0.001
	No	371 (1.1)				
History of cancer, *n* (%)	Yes	75 (1.5)	1.35 (1.05–1.73)	0.019		
	No	366 (1.1)				
Prior hospitalization for bleeding, *n* (%)	Yes	95 (1.5)	1.35 (1.08–1.70)	0.001		
	No	346 (1.1)				
Chronic respiratory disease, *n* (%)	Yes	49 (1.7)	1.49 (1.10–2.01)	0.009		
	No	392 (1.2)				
Critical preoperative state, *n* (%)	Yes	32 (4.0)	3.65 (2.53–5.27)	<0.001	2.55 (1.73–3.76)	<0.001
	No	405 (1.1)				
Angina at rest, requiring intravenous nitrates, *n* (%)	Yes	78 (2.2)	2.04 (1.60–2.62)	<0.001	1.67 (1.28–2.17)	<0.001
	No	360 (1.1)				

CI: confidence interval; OR: odds ratio; SD: standard deviation.

### Associations between stroke and all-cause mortality

#### Early mortality

Unadjusted mortality during the first postoperative 30 days was 16.3% (72/441) in the stroke group and 1.3% (455/36 457) in the no-stroke group (*P* < 0.001), Table 3. After multi-adjustment, the HR for mortality in stroke patients was 7.29 (95% CI 5.58–9.54), *P* < 0.001. For the period from 30 days to 1 year, the unadjusted mortality was 12.6% (40/329) vs 1.8% (639/35 401) and the adjusted HR (aHR) 3.67 (95% CI 2.61–5.15) (*P* < 0.001), Table 3. The proportional hazards assumption was not fulfilled for these 2 intervals, and *P*-values for interaction with time were 0.0024 and <0.0001, respectively. To study change in HR over time, analyses were performed for the following time periods: first 7 days, aHR 4.40 (95% CI 2.70–7.17), 7–30 days, aHR 9.96 (95% CI 7.22–13.74), 30 days to 3 months, aHR 6.91 (95% CI 4.43–10.78) and 3 months to 1 year, aHR 2.01 (95% CI 1.15–3.49).

**Table 3:** The crude mortality and adjusted mortality risk in coronary artery bypass grafting patients with and without perioperative stroke

	Stroke patients, % (*n*)	No-stroke patients, % (*n*)	Hazard ratio (95% CI)	*P*-Value
Mortality overall	50.8 (224/441)	19.5 (6990/36 457)	2.30 (2.00–2.64)	<0.0001
Mortality within 30 days	16.3 (72/441)	1.3 (455/36 457)	7.29 (5.58–9.54)	<0.0001
Mortality from 30 days to 1 year	12.6 (40/329)	1.8 (639/35 401)	3.67 (2.61–5.15)	<0.0001
Mortality from 1 to 5 years	13.1 (38/291)	7.7 (2530/32 871)	1.10 (0.79–1.52)	0.57
Mortality from 5 years	34.1 (74/224)	11.4 (3366/29 505)	1.76 (1.39–2.23)	<0.0001

The models have been adjusted for sex, age, history of myocardial infarction, previous stroke, previous transient ischemic attack, diabetes, hypertension, heart failure, atrial fibrillation, renal failure, peripheral vascular disease, history of cancer, hospitalization for bleeding, chronic respiratory disease, acute coronary syndrome, mechanical circulatory assist preoperatively, prior cardiac surgery, critical preoperative state and angina at rest.

CI: confidence interval.

#### Mid-term and long-term mortality

**Figure 2:**
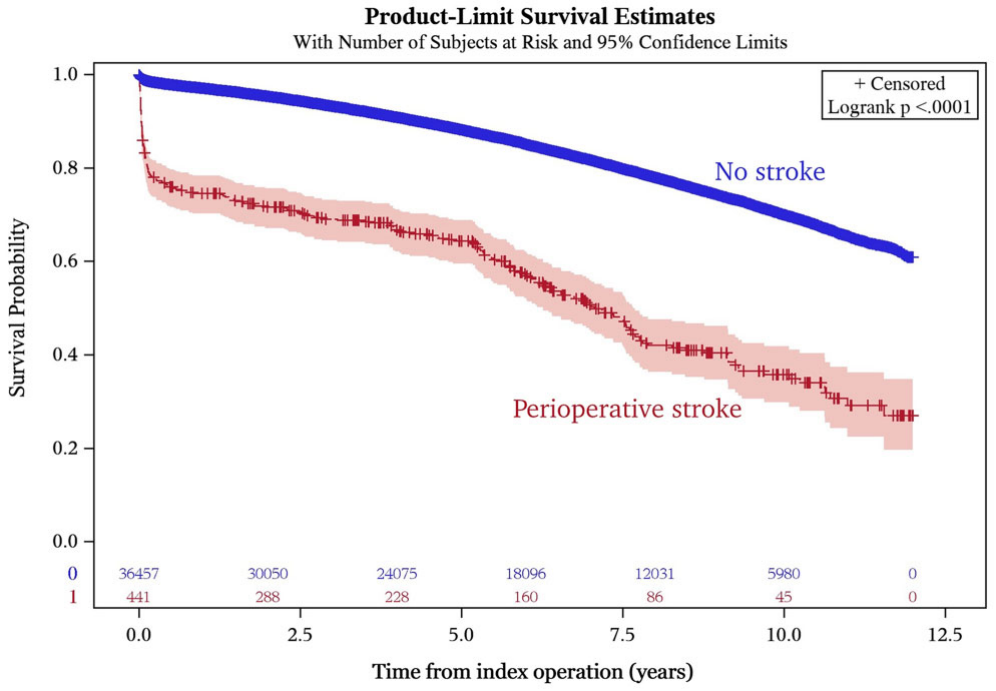
Cumulative survival in coronary artery bypass grafting patients with and without perioperative stroke.

During the total follow-up period, including both early and late mortality, 224/441 (50.8%) of the stroke patients and 6990/36 457 (19.5%) of the no-stroke patients died. The cumulative mortality in stroke and no-stroke patients is depicted in Fig. 2. Stroke patients had a higher overall mortality risk, including both early and late mortality, during follow-up [aHR 2.30 (95% CI 2.00–2.64), *P* < 0.001].

For the period 1–5 years postoperatively, the unadjusted mortality was 13.1% (38/291) in the stroke group and 7.7% (2 530/32 871) in the no-stroke group (*P* < 0.001), Table 3. After multi-adjustment, the HR for mortality in stroke patients was during this period 1.10 (95% CI 0.79–1.52) (*P* = 0.57). For the period after 5 years, the unadjusted mortality was 34.1% (74/224) vs 11.4% (3 366/29 505) and the aHR 1.76 (95% CI 1.39–2.23) (*P* < 0.001), Table 3.

## DISCUSSION

The main findings in this nationwide observational cohort study were that the incidence of perioperative stroke after CABG remained largely unchanged over time and that patients with perioperative stroke had a markedly higher adjusted risk of death early after surgery. The increased mortality risk was sustained up to 12 years after the index operation.

The incidence of stroke after CABG in our large study cohort was 1.2%, which is comparable or lower than in most previous studies on the subject. In Gaudino *et al.*’s [5] recent large meta-analysis in mixed cardiac surgery patients, the incidence of perioperative stroke was 2.0%. In a systematic review of CABG patients, the incidence varied between 1.1% and 5.7% in the included studies [6] while a very large registry study from the USA, compromising over 668 000 CABG patients reported an incidence of 1.9% [1].

The unadjusted incidence of perioperative stroke in our study remained largely unchanged during the period 2006–2017. In contrast to most previous reports, we also analysed the adjusted incidence over time and found that, when adjusted for factors associated with stroke risk, there was a statistically significant reduction in the perioperative stroke risk over time. However, this information should not be overvalued since the reduction was small (relative reduction ∼3% per year). This is a very marginal reduction given the 1.2% absolute annual incidence and hence not clinically meaningful.

In the present study, we also identified preoperative risk factors associated with perioperative stroke. The majority of these have been previously identified and were thus confirmed in the present large cohort study. It can be noted that the importance of the patient’s preoperative condition was strong, as also age, previous stroke and the presence of peripheral arterial disease. Female sex was also identified as a strong independent factor, which is in accordance with previous studies [2, 15]. There was a significant interaction with age for female sex with relatively higher risk for women <70 years old, but no interaction with previous stroke, previous heart surgery or preoperative critical state.

Thirty-day mortality was 13-fold higher (16.3% vs 1.3%) in patients with perioperative stroke. This confirms the extremely high mortality in perioperative stroke previously reported [4, 15–17]. Similar figures were reported in Gaudino *et al.*’s [5] study in mixed cardiac surgery patients where there was a 12-fold difference. After adjustment in the present study for the increased prevalence of risk factors in patients that developed stroke, the OR for mortality in CABG patients with perioperative stroke remained markedly increased (adjusted OR 7.3). This points to the importance of addressing this potential complication in CABG patients and that even a small reduction in the incidence of perioperative stroke would save lives. Perioperative stroke was also associated with increased long-term mortality risk, also when patients who died early were excluded. The adjusted long-term mortality risk after CABG has not been as thoroughly investigated previously as the early risk but there are some reports with similar findings [5, 18–20]. The increased long-term risk indicates that long-term surveillance and secondary prevention measures are of utmost importance in patients who develop perioperative stroke.

### Limitations and strengths

This study has both strengths and limitations. The main limitation of this study is the retrospective nature of the study. While the data are collected prospectively, eliminating recall bias, there is always the risk of selection bias, as well as residual confounding. Another limitation of the study is the inability of our dataset to further define the severity of the stroke and its impact on the patients. Information about the proportion of stroke patients who underwent imaging is lacking in the registries. It should also be noted that we did not investigate the association between intraoperative factors and perioperative stroke. Factors such as cardiopulmonary bypass time and type of surgery have previously been shown to be associated with the risk for perioperative stroke in cardiac surgery patients [3, 4, 16]. One may argue that we should have included also perioperative factors in our statistical models but it adds methodological complexity to mix preoperative factors, known at the time of the decision to operate, with intraoperative factors, unknown at the time of the decision. One of the prespecified aims of the present study was to identify preoperative factors associated with perioperative stroke. This aim was included to gain information that could support the heart team in the discussions on the risk for perioperative stroke. Including also perioperative factors may blur the picture.

The strengths include the large study population in a real-world setting, the long and extensive follow-up, the nationwide coverage with a high degree of data completeness in validated mandatory national registries and databases and a primary end point (all-cause mortality) not subject to bias.

## CONCLUSIONS

There was a marginal decrease in the incidence of perioperative stroke over the study period. Patients with perioperative stroke have a markedly higher adjusted mortality risk of death early after surgery. The risk declines over time but remains higher during the entire follow-up period. Efforts to reduce the incidence of perioperative stroke are essential.

## SUPPLEMENTARY MATERIAL

Supplementary material is available at *EJCTS* online.

## Funding

This work was supported by grants from the Swedish Heart-Lung Foundation (20180560 to Anders Jeppsson and 201604 to Susanne J. Nielsen), the Swedish state under the agreement between the Swedish government and the county councils concerning economic support of research and education of doctors (ALF agreement) (ALFGBG-725131 to Anders Jeppsson), Västra Götaland Region (VGFOUREG-847811 and VGFOUREG-665591 to Anders Jeppsson) and Family Nils Winberg’s Foundation. The supporting bodies had no influence on the analysis and interpretation of data, on the writing of the report or on the decision to submit the paper for publication.

**Conflict of interest:** Anders Jeppsson has received fees for consultancy or lectures from Werfen, Boehringer-Ingelheim, Portola, Baxter and LFB, all unrelated to the present work. The remaining authors have nothing to disclose.

### Author contributions

**Kristjan Jonsson:** Conceptualization; Data curation; Formal analysis; Investigation; Methodology; Validation; Visualization; Writing—original draft; Writing—review & editing. **Mikael Barbu:** Conceptualization; Formal analysis; Writing—review & editing. **Susanne J. Nielsen:** Conceptualization; Data curation; Formal analysis; Investigation; Methodology; Writing—original draft; Writing—review & editing. **Brynhildur Hafsteinsdottir:** Formal analysis; Methodology; Writing—review & editing. **Tomas Gudbjartsson:** Conceptualization; Writing—review & editing. **Elin M. Jensen:** Conceptualization; Writing—review & editing. **Martin Silverborn:** Conceptualization; Formal analysis; Writing—review & editing. **Anders Jeppsson:** Conceptualization; Data curation; Formal analysis; Methodology; Project administration; Supervision; Writing—original draft; Writing—review & editing.

### Reviewer information

European Journal of Cardio-Thoracic Surgery thanks Nadejda Monsefi, Takashi Murashita and the other, anonymous reviewer(s) for their contribution to the peer review process of this article.

## Supplementary Material

ezac025_Supplementary_Data

## Notes

Presented at the 35th Annual Meeting of the European Association for Cardio-Thoracic Surgery, Barcelona, Spain, 13–16 October 2021.

## ABBREVIATIONS

aHR: Adjusted hazard ratio
CABG: Coronary artery bypass grafting
CHA2DS-VAS: **C**ongestive heart failure, **H**ypertension, **A**ge ≥ 75 years **D**iabetes mellitus, previous **S**troke or transient ischemic attack **V**ascular disease, **A**ge 65–74 years, **S**ex category female
CI: Confidence interval
HR: Hazard ratio
ICD: International Statistical Classification of Diseases system
OR: Odds ratio
SWEDEHEART: Swedish Web System for Enhancement and Development of Evidence-Based care in Heart disease Evaluated According to Recommended Therapies
