# Supplementary material for: Perioperative stroke and survival in coronary artery bypass grafting patients: a SWEDEHEART study
Source: Eur J Cardiothorac Surg. 2022 Jan 25;62(4):ezac025. doi: 10.1093/ejcts/ezac025 (PMC9643741; doi:10.1093/ejcts/ezac025)
Supplement: ezac025_Supplementary_Data [file ezac025_supplementary_data.pdf]

Supplementary appendix to

PERIOPERATIVE STROKE AND SURVIVAL IN CORONARY ARTERY BYPASS GRAFTING PATIENTS:

A SWEDEHEART STUDY

Supplementary table 1.

International Classification of Diseases (ICD) 10, codes used for classification of variables from the Swedish National Patient Register.

**Variables**	**ICD-9 1986-1996**	**ICD-10 1997-2017**
Diabetes	250	E10-E14
Renal failure	584-586	N17-N19
Heart failure	428	I50
Hypertension	401-405	I10-I15
Previous myocardial infarction and acute coronary syndrome	410	I210-I214, I219
Previous stroke	431, 434, 436, 432X	I61, I62, I63, I64, I69
Transient ischemic attack	435, 436	I65, I66, G45
Peripheral artery disease	440-444, 447	I70-174, I77
Chronic pulmonary disease	490-496	J40-J47
History of atrial fibrillation	427D	I48
Hyperlipidemia	-	E78
History of cancer	140-208, C00-C97	C00-C97
Bleeding	280, 282, 285- 287, 362W, 430- 432, 530C, 531- 533, 578A-B, 599H, 626G, 626W, 626X, 719B, 784H, 784W, 786D	D50, D62, D68-D69, H356, H922, I230, I312,I60-I62, I690-I692,I850, I983, J942, K221, K226, K25-K28, K290, K625, K661, K920, K921, K922, M250, N02, N398, N421, N501A, N939, N950, R31, R040-R042, R048, R049
